# Priming the primary motor cortex with transcranial direct current stimulation: Effect on learning the golf putt

**DOI:** 10.1371/journal.pone.0324983

**Published:** 2025-05-28

**Authors:** Bisman Mangat, Luc Tremblay, Joyce L. Chen

**Affiliations:** Faculty of Kinesiology and Physical Education, University of Toronto, Toronto, Ontario, Canada; Tehran University of Medical Sciences, IRAN, ISLAMIC REPUBLIC OF

## Abstract

**Background:**

Priming the primary motor cortex (M1) with transcranial direct current stimulation (tDCS) prior to motor practice modulates post-synaptic activity, thereby impacting learning of a motor skill. This effect has been shown for the acquisition of simple motor skills. It is not clear whether priming tDCS can impact the learning/retention of a more naturalistic motor task.

**Objective/Hypothesis:**

We investigated the effects of priming M1 with tDCS on the performance on a golf putting task. We hypothesized that participants who receive tDCS with the cathode over M1 (C-M1) would show better skill acquisition and retention performance, relative to participants who receive tDCS with the anode over M1 (A-M1) or sham tDCS.

**Methods:**

Thirty-six participants were randomized into three groups: C-M1, A-M1, and sham tDCS. Participants received tDCS (1mA, 20 minutes) prior to practicing golf putting across two days. Performance (error) was measured for each putt. Participants returned on the third day for a retention test.

**Results:**

After accounting for baseline performance, the C-M1 group performed significantly better compared to A-M1 [p = 0.02] and sham tDCS [p = 0.01] at the retention test. There was no difference in retention performance between A-M1 and sham tDCS.

**Conclusion:**

Our findings partially support the Bienenstock-Cooper-Munro rule of metaplasticity. C-M1 tDCS priming enhanced motor learning, while A-M1 tDCS priming had no effect, relative to sham.

## 1. Introduction

Practice is required to learn a motor skill such as those involved in playing a sport or musical instrument. Motor learning refers to processes that lead to a relatively permanent change in performance, as a result of practice or experience [1]. One neural mechanism that supports motor learning involves the strengthening and/or weakening of synapses, known as long-term potentiation (LTP) and long-term depression (LTD), respectively [2]. According to the Bienenstock-Cooper-Munro (BCM) rule of metaplasticity, the ability to induce LTP or LTD depends on previous synaptic activity [3]. A history of high synaptic activity increases the threshold for LTP, making it harder to induce synaptic strengthening to support learning. In contrast, a history of low synaptic activity decreases the threshold for LTD, making it easier to induce synaptic strengthening to support learning. This sliding threshold aims to keep synaptic activity within an optimal physiological range, while preventing over-excitation or inhibition [4].

Transcranial direct current stimulation (tDCS) is a form of non-invasive brain stimulation that can be applied to modulate cortical excitability and influence the synaptic modification threshold described by the BCM rule [5–7]. Depending on the polarity of stimulation, tDCS can modify cortical excitability in ways consistent with the BCM rule. Specifically, at the neurophysiological level, stimulation with the anode over M1 (A-M1 tDCS) relatively increases corticospinal excitability (CSE), thereby raising synaptic activity and the threshold for inducing further LTP-like plasticity [5]. Conversely, stimulation with the cathode over M1 (C-M1 tDCS) relatively decreases CSE, thereby reducing synaptic activity and lowering the threshold for subsequent LTP induction [8]. Therefore, we investigated whether tDCS applied in a ‘priming’ fashion can facilitate motor skill learning.

The application of tDCS *prior to* motor practice is thought to prime neurons to facilitate learning [7]. Some studies show the expected behavioural result where A-M1 tDCS priming leads to worse performance [9,10] or has no effect on performance [11] on the serial reaction time task (SRTT). However, others show that A-M1 tDCS priming results in better performance on a visuomotor tracking task [12]. Similarly, some studies show the expected result where C-M1 tDCS leads to better performance on a visuomotor tracking task [12]. However, others show that C-M1 tDCS priming results in either worse performance [10] or has no effect on performance [11] on the SRTT. Taken together, findings in support of the BCM rule are inconsistent. However, a potential limitation of this prior work is that motor learning was not tested, which is typically assessed with a retention test (cf. performance during acquisition). Retention tests are completed at least 24 hours to several weeks/months after skill acquisition [1,13]. It is important to address the acquisition-learning distinction as effects of practice during skill acquisition may impair performance but enhance skill retention, or alternatively, augment performance but impair skill retention [13]. Therefore, our study will aim to dissociate effects of priming tDCS on skill acquisition and retention following 2 days of practice. Participants completed two days of practice, as previous work has demonstrated that a single session of tDCS is not sufficient for lasting effects on motor performance [14]. Further, we combine tDCS with a more naturalistic motor task, golf putting, as opposed to traditional laboratory-based motor tasks (e.g., SRTT). We selected golf putting because it is a closed motor skill with well-defined parameters and has been previously used in tDCS research to assess motor performance [15,16].

The objective of our study was to investigate the effects of priming tDCS to M1 on the acquisition and retention (i.e., learning) of a golf putting task. We hypothesized that participants who receive C-M1 tDCS will show the greatest improvements in performance across blocks of acquisition, and better retention, as compared to participants who receive A-M1 tDCS or sham tDCS. An exploratory aim was to investigate the effects of priming tDCS to M1 on the transfer or generalizability of performance to different contexts.

## 2. Methods

### 2.1. Participants

We recruited thirty-six right-handed participants, as per the short form of the Edinburgh Handedness Inventory [17], between 18 and 44 years old. Exclusion criteria were contraindications to tDCS [18], history of neurologic or psychiatric disorder [19], use of medication(s) that potentially interact with tDCS [20], upper body injury that may hinder the ability to perform the motor task, and/or prior experience in golf, putting, or ball-and-stick sports (e.g., lacrosse, hockey, tennis). The study was approved by the University of Toronto Research Ethics Board (REB: 38530). All participants provided written informed consent prior to participation.

### 2.2. Study design

**Fig 1:**
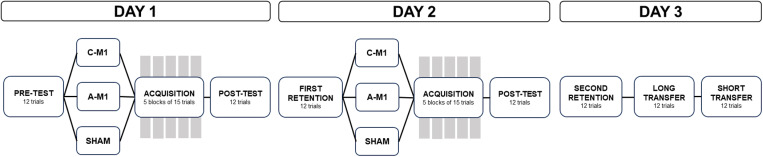
Study design. On the first session, participants completed a pre-test, received transcranial direct current stimulation (tDCS), trained on the golf putting task, and completed a post-test. On the second session, participants completed a retention test (first retention), received tDCS, trained on the golf putting task, and completed another post-test. On the third session, participants completed a retention test (second retention) and two transfer tests on the golf putting task. Questionnaires were completed throughout the three sessions (see Section 2.5).

Participants attended three sessions, held on consecutive days approximately 24-hours apart (Fig 1). Participants were randomized into one of three tDCS groups: A-M1, C-M1, or sham (see section 2.3). On session 1, they performed a pre-test on the golf putting task (see section 2.4), received tDCS, completed acquisition trials, and then a post-test on the task. On session 2, participants performed a retention test (first retention), followed by tDCS, acquisition trials, and a post-test on the task. Session 3 entailed performance on a retention test (second retention) and two transfer tests (see section 2.4). Questionnaires were administered before and after each session to understand how different factors may affect performance and to probe experiences associated with tDCS [20] (see section 2.5).

### 2.3. Transcranial direct current stimulation

This was a double-blind study with both the researcher and the participants unaware of which stimulation group the participant was in. A research assistant not involved in the collection of data pseudorandomized participants into one of three groups to ensure equal numbers of participants in each group. Further, the research assistant generated unique study codes the researcher inputted to the tDCS device.

tDCS was delivered through the NeuroConn DC Stimulator Plus (Neurocare, Germany). Two electrodes, each 5 cm x 5 cm were placed into sponges saturated with 12 mL of saline solution each and secured to the head using rubber bands. With the University of Toronto Varsity Golf Coach, the shoulder joint of the dominant side of the body was identified as important for golf putting. Thus, the targeted region was C1 on the 10–20 Electroencephalogram (EEG) system as it may more closely approximate the shoulder representation in the left M1 than C3 (cf. hand representation) [21]. In the A-M1 group, the anode was placed over C1, and the cathode was placed over the contralateral supraorbital region. The opposite placement was employed for the C-M1 group. In the sham group, we applied the A-M1 configuration to half of the participants and the C-M1 configuration to the other half.

To confirm that the electrode placement would result in current flowing through the desired region of left M1, current flow modelling was used. The software package ROAST was used and the methodology described by Evans et al. [22] was followed. A sample MRI structural T1 scan was used to create a model of the e-fields generated in the brain through tDCS (images available in Supplementary Material). This MRI was of the last author of the paper (JLC), obtained for pilot testing. The outputs of the modeling in the MRI scan showed that the current did approximately target the left M1 region.

Both the A-M1 and C-M1 groups received 1mA (current density 0.04mA/cm^2^; total charge 0.048C/cm^2^) of tDCS for 20 minutes. These parameters were selected because 1mA applied for 20 minutes is commonly used in tDCS studies investigating M1 excitability, and has been shown to induce polarity-specific changes in CSE (i.e., increased CSE with A-M1, decreased CSE with C-M1) [23–25]. Lower intensities have been shown to produce weaker or inconsistent effects, while higher intensities (e.g., 2mA) may increase variability in individual responses [23]. Additionally, using this standard protocol allowed us to compare to prior work using similar stimulation protocols [9].

The current was ramped on/off over 30 seconds. In the sham condition, the current was ramped on over 30 seconds, stayed on for 30 seconds, and then was ramped down over 30 seconds and remained off for the remainder of the session. During the 20 minutes of tDCS, all participants were seated in a chair and refrained from moving or speaking except to report discomfort. Following termination of the tDCS, participants completed a questionnaire that inquired about their experiences with the stimulation. This was done before the electrodes were removed to allow time for the sponges to cool down and ensured that the researcher collecting data remained blind to the stimulation condition.

### 2.4. Golf putting task

**Fig 2:**
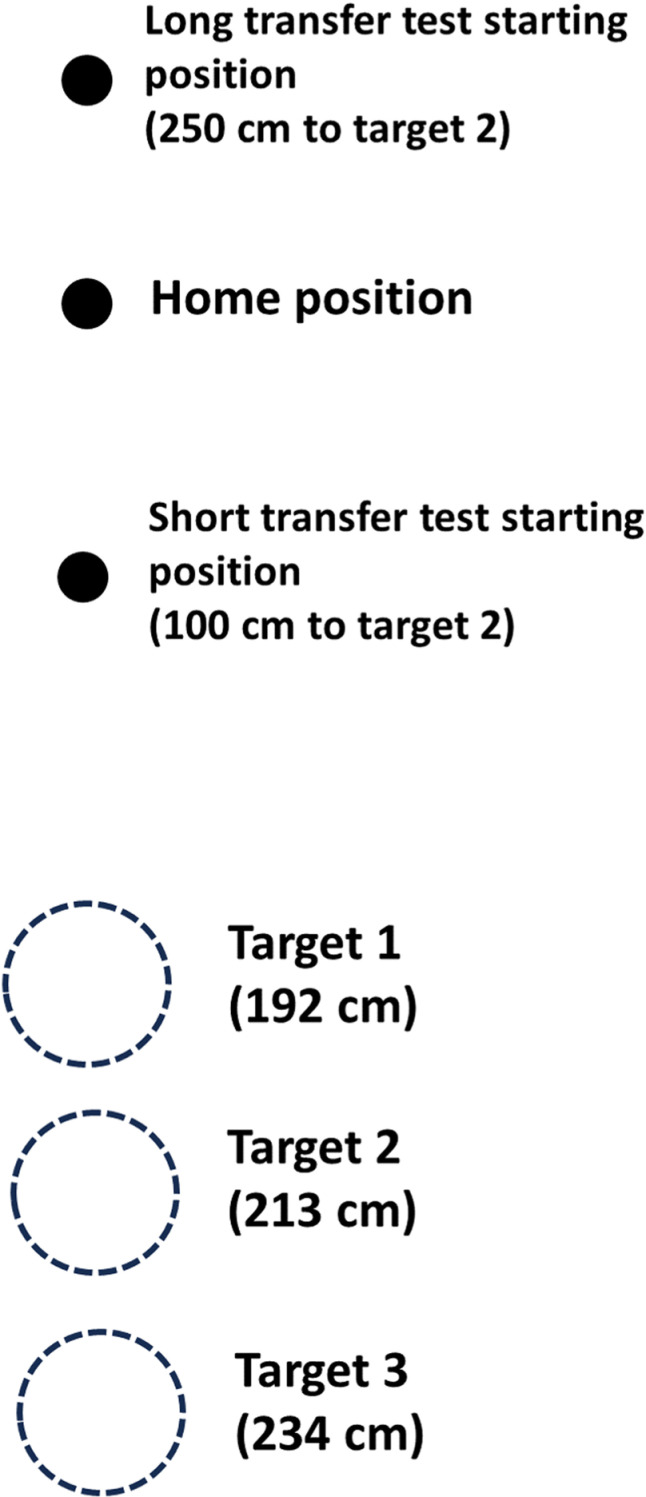
Golf putting green. Figure is not to scale. The golf putting green has three targets. Target 1, Target 2, and Target 3 were 192 cm, 213 cm, and 234 cm from the home position, respectively. Targets were lit up using LEDs. Both transfer tests consisted of 12 trials to Target 2. To achieve this, the starting spot for the ball was moved 250 cm and 100 cm from Target 2 for the long and short transfer tests, respectively.

Approximately 10 minutes after the termination of tDCS, participants engaged in a golf putting task (Fig 2), adopted based on prior work from our group [26]. The objective of the task was to putt a standard golf ball so that it landed within a target using a golf putter. Note, there was no physical hole. Targets were circles on the putting green, illuminated by light-emitting diodes (LEDs). Based on consultations with the University of Toronto Varsity Golf Coach, participants were instructed with the following: 1) stand with feet shoulder width apart with the ball in the centre of your stance; 2) place your dominant hand at the bottom of the grip and your non-dominant hand at the top of the grip; 3) look at the target first, then swing the golf club using your shoulders to hit the ball towards the target.

The putting green was 488 cm long and 122 cm wide, with three targets located at distances of 192 cm (Target 1), 213 cm (Target 2), and 234 cm (Target 3) from the home position to the centre of the target (Fig 2). Each target was 10.8 cm in diameter. Targets were only visible when the LEDs were on, with only one target illuminated per trial. The pre-test, post-test, and retention test consisted of 12 trials to Target 2. Motor skill acquisition consisted of 75 trials on each day (150 trials over two days) to all three targets in a variable order, based on motor learning principles [1]. All participants received the same order of targets for the acquisition trials. Acquisition trials were separated into blocks of 15 trials (5 blocks each day, 10 blocks total over two days). The transfer tests consisted of 12 putts to Target 2. For these transfer tests, participants moved away from the home position, such that novel distances were created relative to Target 2 (long transfer test = 250 cm; short transfer test = 100 cm) (Fig 2).

**Fig 3:**
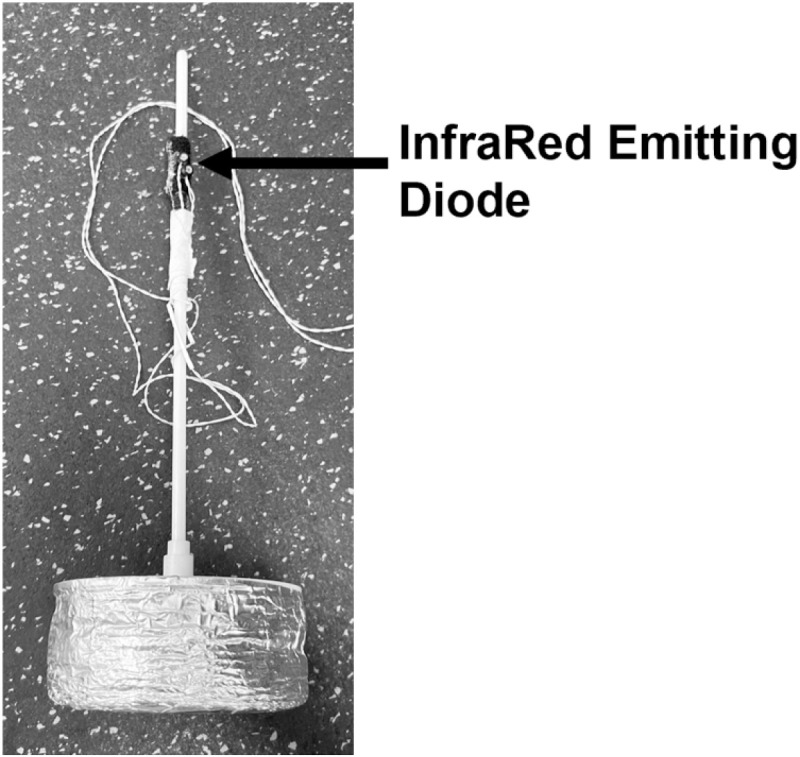
Data collection device. An InfraRed Emitting Diode was connected to a device. Following each putt, the device was placed on top of the ball to collect ball end-point location using the Optotrak motion tracking system.

We controlled the experiment and collected data using MATLAB (The Mathworks Inc., Natrick, MA, USA). We used the Optotrak motion tracking system (Northern Digital Inc., Waterloo, ON, Canada) to record target location and ball end-point location. The Optotrak motion tracking system measures global position in three dimensions (x, y, and z). Data from the ball endpoint position relative to the target hole in the x and y dimension were of relevance and were analyzed. Prior to each session, the location of each target was recorded using an InfraRed Emitting Diode (iRED). Following each putt, once the ball came to a stop, we recorded the position of the ball by placing an iRED marker over the ball using a custom-made device (Fig 3). After the data was collected, the ball was placed back at the home position and the target for the next trial was turned on.

### 2.5. Questionnaires

We administered a baseline state assessment at the beginning of each session to quantify variables that may impact the ability to learn a motor skill and/or affect response to tDCS [20]. We inquired about sleep [27], caffeine intake [28], prior physical activity, motivation [29], and fatigue [30]. Quality of sleep was recorded on a scale of one to five (one = poor, five = excellent). Motivation was recorded on a scale of one to five (one = not motivated, five = very motivated). Fatigue was rated on a scale of one to five (one = no soreness, five = very sore).

We administered a tDCS debrief form following tDCS on sessions 1 and 2. Participants were asked to rate the presence and severity of ten sensations (i.e., itching, tingling, pinching, pain, burning, warmth, fatigue, headache, dizziness, discomfort) on a scale of one to five (one = none, five = strong) [31]. Participants were also asked to guess which tDCS group they thought they were in on a multiple-choice question (anodal, cathodal, or sham).

### 2.6. Data analysis

All statistical analyses were performed in R (version 4.1.2). Data was evaluated for normality (i.e., Shapiro-Wilk) and homogeneity of variance (i.e., Levene). Outliers were identified according to 3 interquartile range for each participant for each block of trials. For the acquisition trials, three participants had block averages that were considered outliers. For the retention trials, one participant had a block average that was considered an outlier. Removing these outliers did not change the pattern of findings at the group level; thus, we present all data. The significance level for all tests was p < 0.05.

We assessed the factor sex (male/female), between the three groups using the Chi-Squared test. We assessed factors age and handedness, between the three groups using a one-way analysis of variance (ANOVA).

The primary dependent measure was radial error, which is defined as the shortest distance from the ball end-point location to the centre of the target position. Error was calculated by subtracting the location of the ball from the location of the target in both the x-axis and the y-axis. Then, the radial error was calculated ($\sqrt{{x\;error}^{2}+{y\;error}^{2}}$). We assessed performance at baseline across the three groups using a one-way ANOVA on radial error. To investigate performance during acquisition, we performed a repeated measures ANOVA with between-subjects factor stimulation type (A-M1, C-M1, sham) and within-subject factors blocks of practice (block 1 to block 5) and day (day 1, day 2). To investigate performance during retention, we performed two one-way analysis of covariance (ANCOVA) on mean radial error with performance on the pre-test as a covariate, separately for the first retention test (day 2) and the second retention test (day 3) (Fig 1). Post-hoc analysis was conducted with pairwise comparisons of estimated marginal means with Tukey’s HSD correction to control for multiple comparisons. To further understand differences in performance, we classified performance as “improved”, “maintained”, or “worsened” on the second retention test compared to the post-test on day 2. To do this, we used a 10% bandwidth. If radial error at the retention test was greater or less than 10% of radial error at the post-test on day 2, performance was classified as worsened or improved, respectively. If radial error at the retention test was within 10% of radial error at the post-test on day 2, then performance was classified as maintained [32]. We selected a 10% bandwidth to avoid classifying small fluctuations in performance across days as an improvement or worsening of performance. We acknowledge that this bandwidth is arbitrary; however, there was no obvious value to choose that would allow us to avoid classifying small fluctuations as improvement. To evaluate if there were differences in the number of participants that improved, maintained, or worsened across groups, we ran a Chi-Squared test. For the transfer data, we performed a one-way ANVOA on radial error, separately for each of the two transfer tests (long, short).

To assess group differences in quality of sleep, motivation, and fatigue, we performed Kruskal-Wallis tests. To assess group differences in the amount of sleep, we performed an ANOVA. To assess group differences in caffeine consumption and exercise, we performed Chi-Squared tests. To assess group differences in the number of participants that reported sensations and correctly guessed the stimulation condition, we performed Chi-Squared tests.

## 3. Results

All raw data are available in the Supplementary Material.

### 3.1. Participants

We tested a convenience sample of 36 participants (N = 23 females, mean age ± standard deviation: 23.6 ± 4.9) to match with previous work on this topic [9]. Participants were pseudo-randomized into three stimulation type: A-M1, C-M1, and sham groups. Baseline demographic information is presented in Table 1. There were no significant differences in sex (p = 0.43), age (p = 0.39), or handedness (p = 0.61) across groups.

**Table 1:** Participant demographics.

	A-M1 (n = 12)	C-M1 (n = 12)	Sham (n = 12)	Test Statistic	p-value
**Sex**	8F, 4M	6F, 6M	9F, 3M	X^2^ (2) = 1.69	0.43
**Age (years), mean (SD)**	23.8 (5.2)	22.1 (2.7)	24.8 (6.0)	F(2, 33) = 0.98	0.39
**Handedness (score), mean (SD)**	100 (0)	97.2 (9.6)	97.6 (8.2)	F(2, 33) = 0.51	0.61

Sex (F = females, M = males), age, and handedness are presented. SD refers to standard deviation. A-M1 refers to tDCS with the anode over M1. C-M1 refers to tDCS with the cathode over M1.

### 3.2. Baseline performance

**Fig 4:**
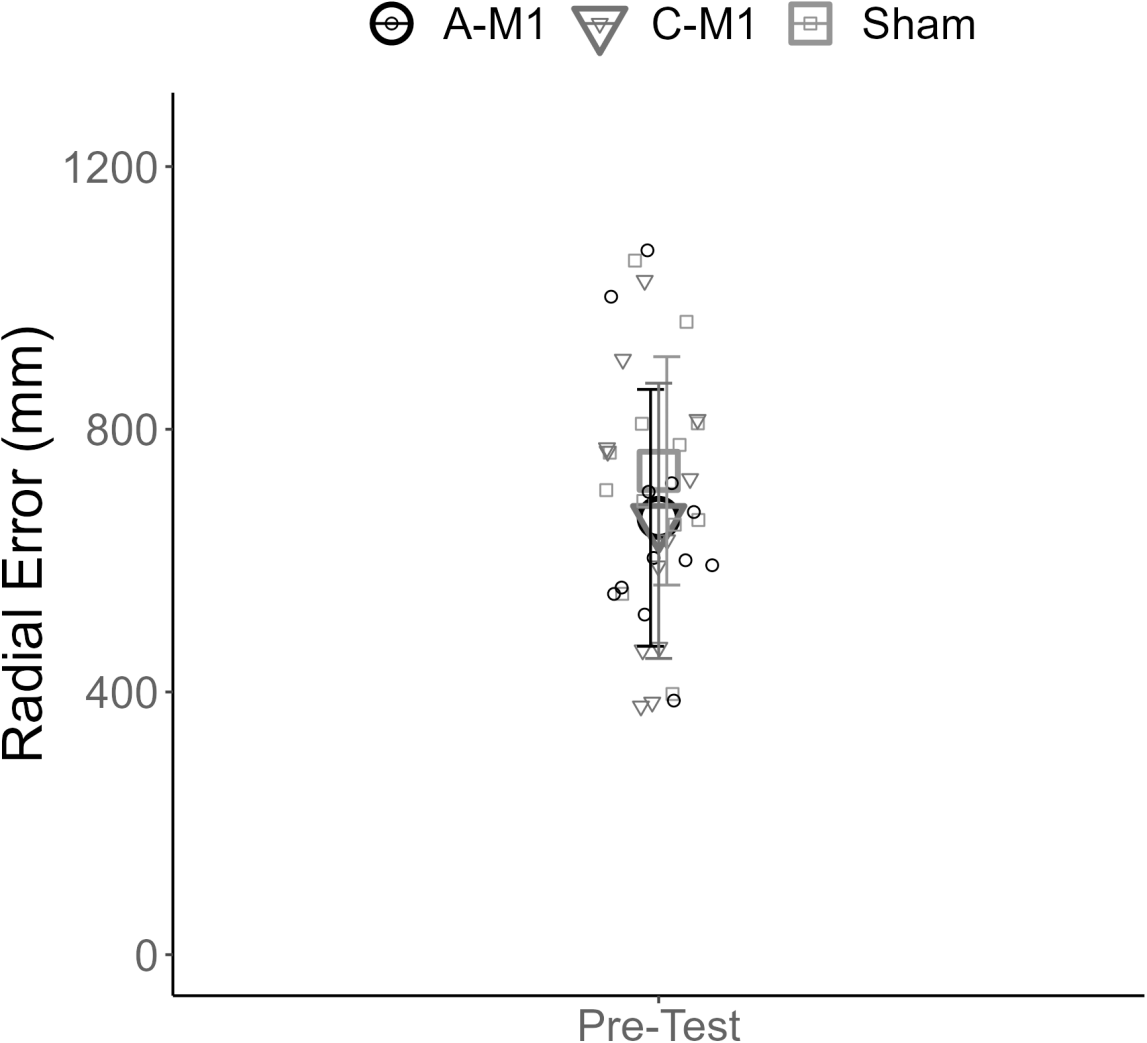
Baseline performance. The x-axis displays the performance block at baseline (pre-test). The y-axis on the figure displays radial error (mm). Group mean radial error is plotted for each of the three groups (A-M1 (○), C-M1(▲), and sham (□)). Individual mean radial error is plotted for each participant (small circles). The error bars reflect standard deviation. A-M1 refers to tDCS with the anode over M1. C-M1 refers to tDCS with the cathode over M1.

There were no differences in radial error across the three stimulation types at baseline (F(2,33) = 0.58; p = 0.56) (Fig 4).

### 3.3. Motor skill acquisition

**Fig 5:**
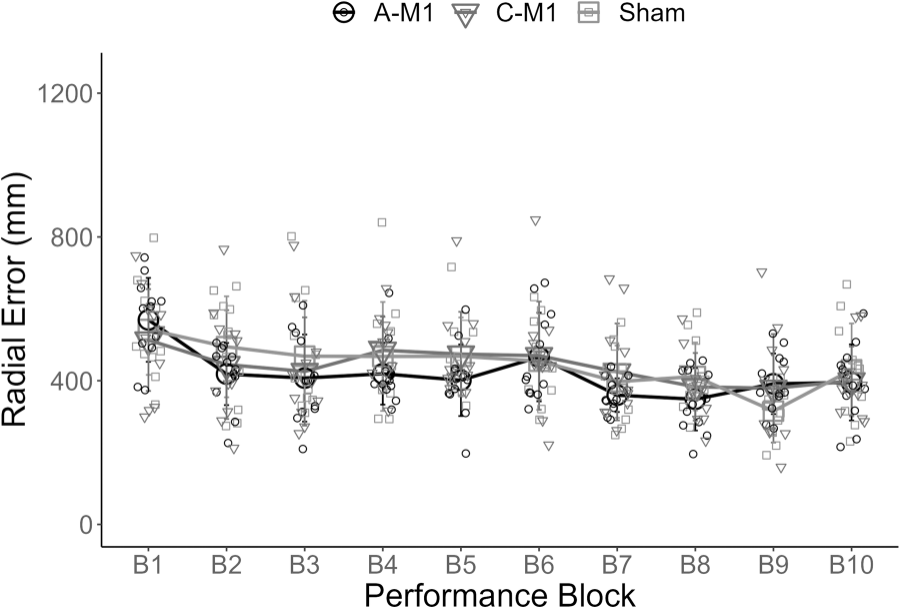
Motor skill acquisition. The x-axis displays performance blocks across day 1 (B1 – B5) and day 2 (B6 – B10) of acquisition. The y-axis displays radial error (mm). Group mean radial error is plotted for each of the three groups (A-M1 (○), C-M1 (▲), and sham (□)). Individual mean radial error is plotted for each participant (small circles). The error bars reflect standard deviation. A-M1 refers to tDCS with the anode over M1. C-M1 refers to tDCS with the cathode over M1.

There was a main effect of practice block; all participants improved their performance (decrease in radial error) (F (4, 132) = 16.94; p < 0.01, η_p_^2 ^= 0.34). There was a main effect of day; performance improved across the two days (F (1, 33) = 31.12; p < 0.01, η_p_^2^ = 0.49). There was no main effect of stimulation group (F (2, 33) = 0.379; p = 0.69), and no interactions for stimulation type x practice block (F (8, 132) = 1.95; p = 0.057), stimulation type x day (F (2, 33) = 0.87, p = 0.43), practice block x day (F (4, 132) = 1.08, p = 0.37), or stimulation type x practice block x day (F (8, 132) = 1.09, p = 0.38) (Fig 5).

### 3.4. Motor skill retention

**Fig 6:**
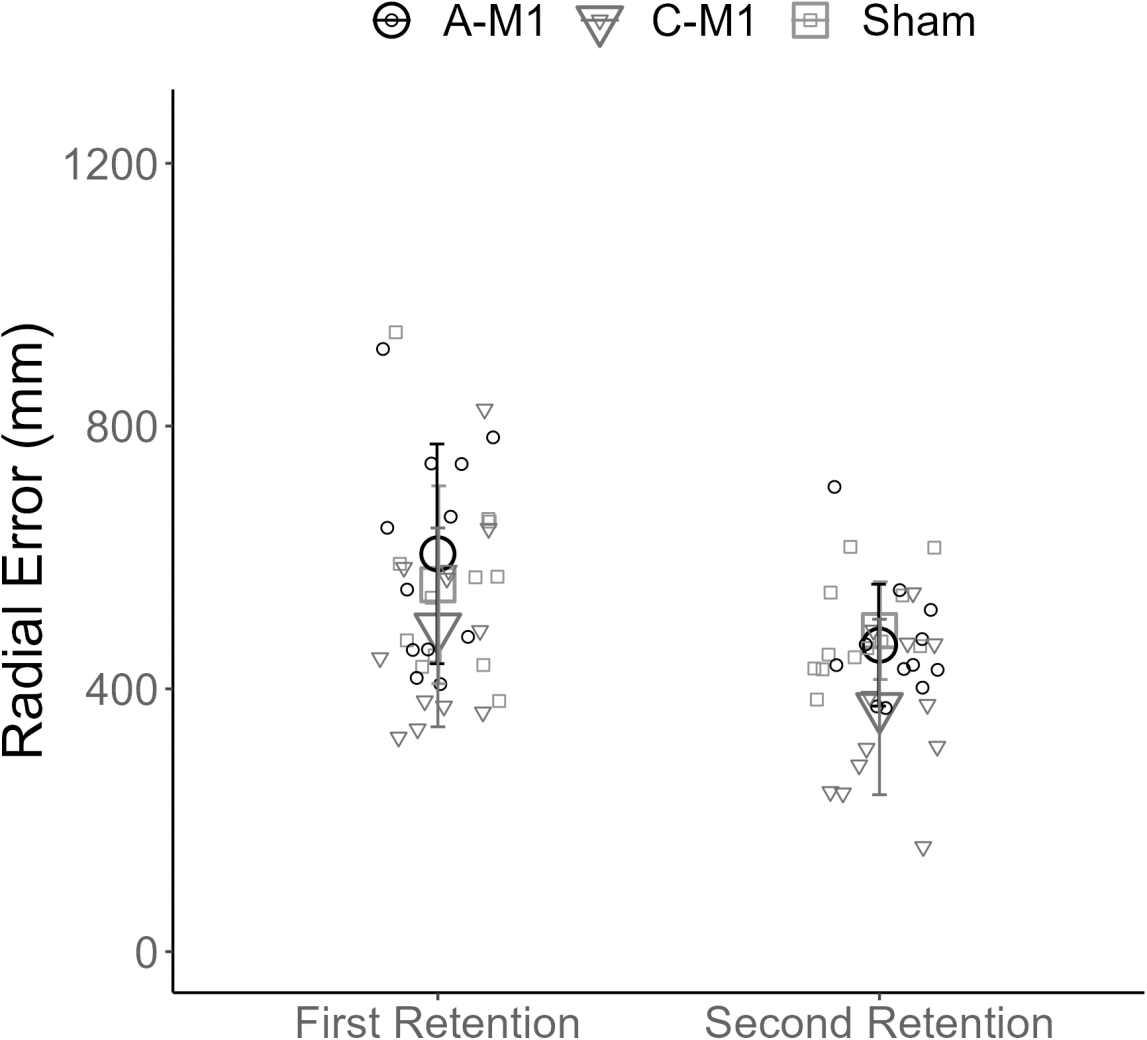
Motor skill retention. The x-axis in the figure displays performance blocks. The y-axis displays radial error (mm). Group mean radial error is plotted for each of the three groups (A-M1 (○), C-M1 (▲), and sham (□)). Individual mean radial error is plotted for each participant (small circles). The error bars reflect standard deviation. A-M1 refers to tDCS with the anode over M1. C-M1 refers to tDCS with the cathode over M1.

On the first retention test, there was no significant effect of group on radial error, after accounting for baseline performance (F (2, 32) = 1.53, p = 0.23). On the second retention test, there was a significant effect of group on radial error, after accounting for baseline performance (F (2, 32) = 5.88, p = 0.007, η_p_^2 ^= 0.27) (Fig 6). Post-hoc pairwise comparisons with Tukey correction demonstrated that radial error was significantly lower in the C-M1 compared to the A-M1 group (t (32) = 2.83, p = 0.02, *d = *1.28) and the sham group (t (32) = -−3.09, p = 0.01, *d = *1.15). There was no significant difference in radial error between the A-M1 and sham group (t (32) = −0.30, p = 0.76).

### 3.5. Change in performance

To further understand group differences at the second retention test, we classified performance as “improved”, “maintained”, or “worsened” compared to the post-test on Day 2. There was a significant difference across groups in the number of participants that improved, maintained, or worsened (χ² (2) = 13.33, p < 0.01). The C-M1 group had a significantly greater number of participants that improved performance from the post-test on day 2 to the second retention test. The A-M1 and sham groups had a greater number of participants that had worse performance from the post-test on day 2 to the second retention test (Table 2).

**Table 2:** Change in performance.

	A-M1 (n = 12)	C-M1 (n = 12)	Sham (n = 12)	Test Statistic	p-value
**Improved**	0	7	2	X^2^(2) = 13.33	0.0098
**Maintained**	4	3	2		
**Worsened**	8	2	8		

Individual performance was classified as improved, maintained, or worsened from the post-test on Day 2 to the retention test on Day 3. Values represent the number of participants. A-M1 refers to tDCS with the anode over M1. C-M1 refers to tDCS with the cathode over M1.

### 3.6. Transfer

**Fig 7:**
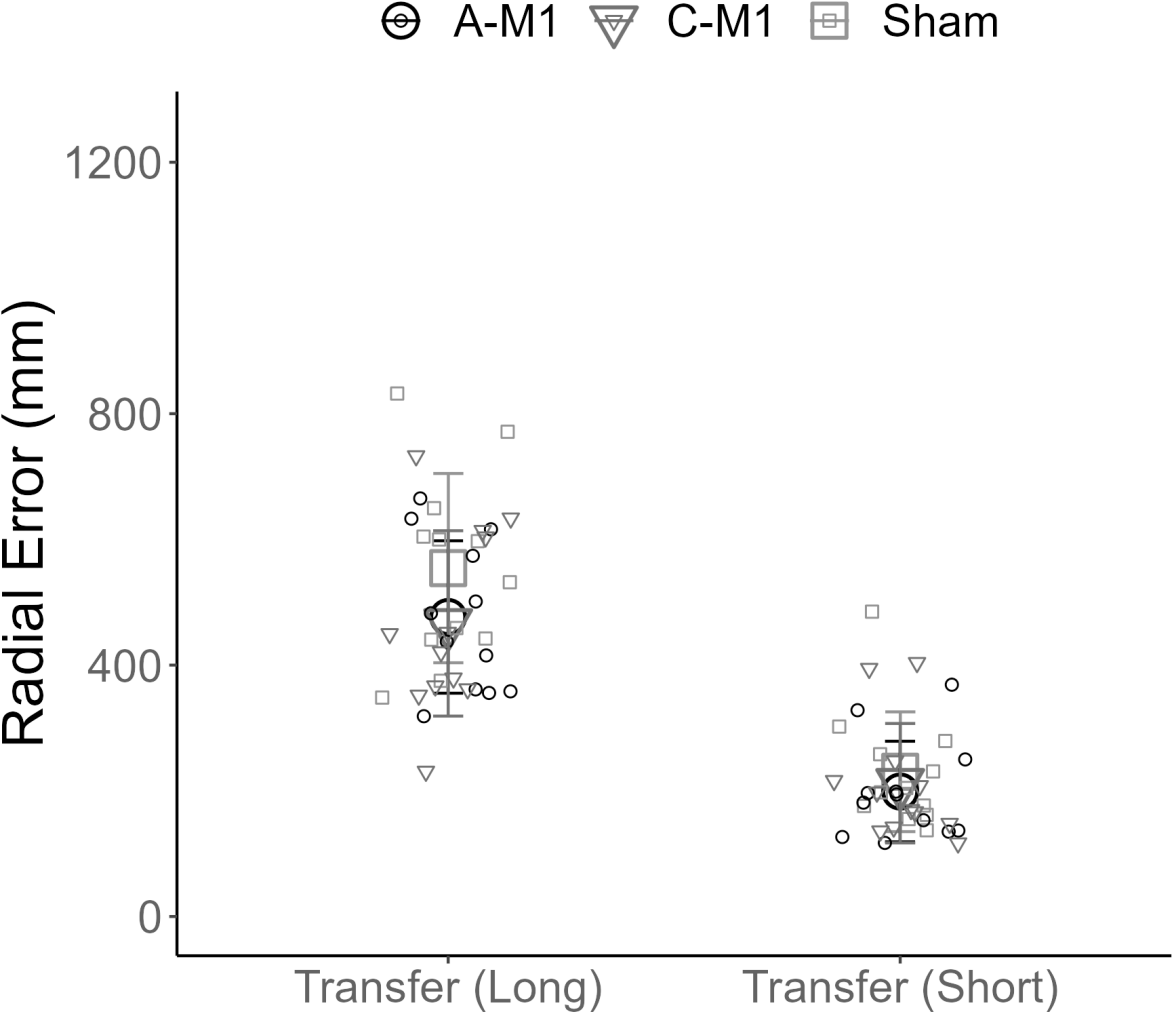
Motor skill transfer. The x-axis displays the performance block. Transfer (long) refers to the long transfer test (250 cm) and Transfer (short) refers to the short transfer test (100 cm). The y axis displays error (mm). Group mean radial error is plotted for each of the three groups (A-M1 (○), C-M1 (▲), and sham (pone.0324983)). Individual mean radial error is plotted for each participant (small circles). The error bars reflect standard deviation. A-M1 refers to tDCS with the anode over M1. C-M1 refers to tDCS with the cathode over M1.

There was no effect of group for radial error on the long transfer test (F (2, 33) = 1.41, p = 0.26) or the short transfer test (F (2, 33) = 0.37, p = 0.69) (Fig 7).

### 3.7. Questionnaires

Baseline state assessment variables are reported in Table 3. There were no significant differences across groups for these measures.

**Table 3:** Baseline state.

	Day 1					Day 2					Day 3				
	**A-M1** **(n = 12)**	**C-M1** **(n = 12)**	**Sham** **(n = 12)**	**Test Statistic**	**p-value**	**A-M1** **(n = 12)**	**C-M1** **(n = 12)**	**Sham** **(n = 12)**	**Test Statistic**	**p-value**	**A-M1** **(n = 12)**	**C-M1** **(n = 12)**	**Sham** **(n = 12)**	**Test Statistic**	**p-value**
**Hours of sleep,** **mean (SD)**	6.7 (1.5)	7.2 (0.7)	7.1 (0.9)	F(2,33) = 0.75	0.48	7.2 (1.1)	6.5 (1)	7.3 (1.2)	F(2,33) = 2.13	0.14	6.9 (1.1)	6.4 (1.6)	7.2 (1.1)	F(2,33) = 1.17	0.32
**Quality of sleep,** **mean (SD)**	3.5 (1.2)	3.8 (0.6)	3.8 (0.5)	*H*(2) = 0.30	0.86	3.8 (1.0)	3.4 (0.5)	3.6 (0.8)	*H*(2) = 1.16	0.56	3.6 (1.2)	3.2 (0.9)	3.4 (1)	*H*(2) = 0.91	0.63
**Number of participants that consumed caffeine prior to start of session (percentage of group)**	6 (50%)	3 (25%)	7 (58%)	*X*^*2*^(2) = 2.93	0.23	6 (50%)	3 (25%)	8 (67%)	*X*^*2*^(2) = 4.24	0.12	5 (42%)	4 (33%)	0 (75%)	*X*^*2*^(2) = 4.67	0.097
**Number of participants that exercised prior to start of session (percentage in group)**	3 (25%)	0 (0%)	5 (42%)	*X*^*2*^(2) = 6.11	0.051	3 (25%)	4 (33%)	2 (17%)	*X*^*2*^(2) = 0.89	0.64	1 (8%)	2 (17%)	3 (25%)	*X*^*2*^(2) = 1.20	0.55
**Motivation,** **mean (SD)**	4.4 (0.7)	4.3 (0.7)	4.6 (0.5)	*H*(2) = 0.70	0.71	4.6 (0.5)	4.3 (0.7)	4.4 (0.7)	*H*(2) = 0.70	0.71	4.5 (0.8)	4.3 (0.5)	4.3 (0.8)	*H*(2) = 1.08	0.58
**Fatigue,** **mean (SD)**	1.5 (0.8)	1.4 (0.5)	1.3 (0.5)	*H*(2) = 0.12	0.94	1.4 (0.8)	1.8 (1)	1.5 (1)	*H*(2) = 1.09	0.58	1.6 (0.8)	1.7 (1.1)	1.3 (0.6)	*H*(2) = 1.09	0.58

Hours of sleep was recorded as the total number of hours slept. Quality of sleep was recorded on a scale of one to five (one  = poor, five = excellent). Motivation was recorded on a scale of one to five (one = not motivated, five = very motivated). Fatigue was rated on a scale of one to five (one = no soreness, five = very sore). SD refers to standard deviation. A-M1 refers to tDCS with the anode over M1. C-M1 refers to tDCS with the cathode over M1.

### 3.8. Sensations associated with tDCS and blinding

The presence and severity of sensations reported after tDCS are reported in Table 4. There were significantly more participants that reported feeling tingling (χ² (2) = 6.11; p = 0.047) and discomfort (χ² (2) = 6.91; p = 0.031) in the C-M1 group, relative to the A-M1 and sham groups on Day 1. The number of participants that reported other sensations did not differ across groups. Further, the number of participants that reported feeling each of the sensations did not differ on Day 2 across groups (Table 4). Statistics were not performed on data associated with the severity of sensations.

**Table 4:** Sensations reported after tDCS.

	Day 1					Day 2				
	**A-M1** **(n = 12)**	**C-M1** **(n = 12)**	**Sham** **(n = 12)**	**Test Statistic**	**p-value**	**A-M1** **(n = 12)**	**C-M1** **(n = 12)**	**Sham** **(n = 12)**	**Test Statistic**	**p-value**
Number of participants that reported feeling sensation (percentage in group), mean rating (SD)										
**Itching**	8 (67%) 1.1 (1.1)	11 (92%) 1.8 (1.3)	7 (58%) 0.8 (0.9)	X^2^(2) = 3.60	0.16	6 (50%) 0.7 (0.8)	12 (100%) 1.8 (1.1)	7 (58%) 1.1 (1)	X^2^(2) = 5.25	0.072
**Tingling**	7 (58%) 0.8 (0.8)	12 (100%) 1.2 (0.4)	9 (75%) 1.3 (1)	X^2^(2) = 6.11	0.047^*^	8 (67%) 0.7 (0.5)	11 (92%) 1.4 (0.8)	10 (83%) 1.6 (1)	X^2^(2) = 2.48	0.29
**Pinching**	3 (25%) 0.3 (0.5)	5 (42%) 0.7 (0.9)	3 (25%) 0.4 (0.8)	X^2^(2) = 1.05	0.59	2 (17%) 0.3 (0.6)	7 (58%) 0.9 (0.9)	5 (42%) 0.8 (1)	X^2^(2) = 4.44	0.11
**Pain**	1 (8%) 0.08 (0.3)	6 (50%) 0.7 (0.8)	5 (42%) 0.6 (0.8)	X^2^(2) = 5.25	0.72	1 (8%) 0.08 (0.3)	5 (42%) 0.4 (0.5)	4 (33%) 0.7 (1.2)	X^2^(2) = 3.60	0.16
**Burning**	6 (50%) 0.6 (0.7)	5 (42%) 0.7 (1)	6 (50%) 0.8 (0.9)	X^2^(2) = 0.22	0.89	6 (50%) 0.6 (0.7)	4 (33%) 0.4 (0.7)	4 (33%) 0.8 (1.1)	X^2^(2) = 0.93	0.63
**Warmth**	5 (42%) 0.5 (0.7)	7 (58%) 0.7 (0.7)	8 (67%) 1 (1)	X^2^(2) = 1.58	0.45	3 (25%) 0.4 (0.7)	4 (33%) 0.3 (0.5)	5 (42%) 0.6 (0.8)	X^2^(2) = 0.75	0.69
**Fatigue**	4 (33%) 0.8 (1.3)	6 (50%) 1.1 (1.4)	3 (25%) 0.5 (1)	X^2^(2) = 1.69	0.43	1 (8%) 0.3 (0.9)	5 (42%) 0.8 (1.1)	5 (42%) 0.7 (1)	X^2^(2) = 4.19	0.12
**Headache**	3 (25%) 0.4 (0.9)	4 (33%) 0.4 (0.7)	3 (25%) 0.4 (0.9)	X^2^(2) = 0.28	0.87	2 (17%) 0.2 (0.9)	1 (8%) 0.08 (0.3)	3 (25%) 0.5 (0.9)	X^2^(2) = 1.20	0.55
**Dizziness**	1 (8%) 0.3 (0.9)	3 (25%) 0.3 (0.5)	2 (17%) 0.3 (0.9)	X^2^(2) = 1.20	0.55	0 (0%) 0 (0)	2 (17%) 0.2 (0.4)	1 (8%) 0.08 (0.3)	X^2^(2) = 2.18	0.33
**Discomfort**	4 (33%) 0.4 (0.7)	10 (84%) 0.9 (0.5)	5 (42%) 0.6 (0.8)	X^2^(2) = 6.91	0.031^*^	3 (25%) 0.3 (0.5)	8 (67%) 0.8 (0.6)	5 (42%) 0.6 (0.9)	X^2^(2) = 4.28	0.12
Number of participants that correctly guessed stimulation group, number in each group (percentage)										
	4 (33%)	9 (75%)	1 (8%)	X^2^(2) = 11.45	0.003^*^	3 (25%)	7 (58%)	3 (25%)	X^2^(2) = 3.85	0.14

Participants were asked to rate the presence and severity of sensations on a scale from one to five (1 = none, 5 = strong). SD refers to standard deviation. A-M1 refers to tDCS with the anode over M1. C-M1 refers to tDCS with the cathode over M1. Statistics are reported for data associated with the presence of sensations. ^*^indicate significance at p < 0.05.

The number of participants that correctly guessed the stimulation group are reported in Table 4. There were significantly more participants that correctly guessed being in the C-M1 group on Day 1, relative to participants that correctly guessed being in the A-M1 or sham groups (χ²(2) = 11.45, p = 0.0033). There was no difference in the number of participants that correctly guessed the stimulation group on Day 2 (χ²(2) = 3.85, p = 0.14).

## 4. Discussion

Our hypotheses were partially supported. There was no difference in acquisition performance across the three tDCS groups. However, we found priming with C-M1 tDCS led to better performance on the second retention test, compared to priming with A-M1 tDCS and sham tDCS.

There was no difference in performance across groups during skill acquisition. Our findings contrast with previous priming studies where A-M1 tDCS led to worse [9] and C-M1 tDCS led to better [12] acquisition performance. A potential reason for this result may be due to the different tasks and outcome measures used between the studies. While we looked at accuracy on a golf putting task, others investigated reaction time on a sequence learning task [9] and accuracy on a visuomotor tracking test [12]. Another speculation is that tDCS to M1 may not have influenced performance because M1 may not have been involved during the early acquisition of golf putting. The initial stages of skill acquisition typically involve cognitive resources that are linked to neural activity in the prefrontal and parietal cortices [33,34]. It may be possible that golf putting requires a greater degree of cognitive involvement because participants must learn to regulate variables such as force, speed, and accuracy when manipulating the putter to hit the ball, coordinating their entire upper limb. This is in relative contrast to a sequence learning task, where participants are asked to press a button as quickly as possible in response to a signal without regulation of force or manipulating a putter.

In contrast, M1 may play a bigger role in the consolidation (i.e., retention) of gains made during acquisition of a relatively complex task [35]. This could explain why we found a significant effect in retention but not acquisition performance.

To the best of our knowledge, we are the first to evaluate the effects of tDCS priming on motor skill learning per se, due to the inclusion of retention and transfer tests [36]. We found that the group that received C-M1 tDCS priming had better performance at the second retention test, relative to both the A-M1 and sham groups. Further, a greater number of participants in the C-M1 group improved performance from the end of practice on day 2 to the second retention test. In contrast, the A-M1 and sham groups had a greater number of participants who had worse performance. These findings were only observed for the second retention test, likely because a single session of tDCS is insufficient [37]. Together, our finding partially supports the BCM rule of metaplasticity, which suggests that low synaptic activity reduces the threshold to induce LTP. By applying C-M1, we lowered excitability in M1. When we followed that up with motor practice, theoretically, participants were more easily able to induce LTP-like processes to learn the task. It may be possible that the lack of acquisition effects may be due to tDCS impacting consolidation and not immediate performance improvements, as seen in previous work [14,38]. It may also be possible that the golf putting task may not have masked the subtle differences in acquisition performance across groups due to the novelty of the task and the observed large performance improvements across blocks for all groups.

Our findings did not support the hypothesis that priming with A-M1 tDCS would worsen motor skill retention. This may be because our parameters for A-M1 tDCS may not have been optimized to induce homeostatic mechanisms and therefore, impact performance on a golf putting task. Prior work has demonstrated non-linear effects of polarity on CSE. For example, while a specific set of parameters may modulate CSE for A-M1 tDCS, it may or may not do the same for C-M1 tDCS [39,40]. Perhaps a greater duration of A-M1 tDCS may have been required to increase synaptic activity to a point where it would lead to a performance decrement. To investigate this further, future studies should integrate measures of CSE into the paradigm.

There were no significant differences across groups on the two transfer tests. All participants did well on the short transfer test. It may be possible that this short putt was too easy and perhaps, a ceiling effect, such that participants reached a level where their performance could not improve any more, may have been reached. Performance on the long transfer test was better than the pre-test for all three groups, suggesting that participants were able to transfer. However, there were no differences across the three groups. One potential reason for this may be that M1 may not primarily be involved in the transfer of performance. Siedler (2010) suggested that the transfer of learning may rely on the cerebellum [41]. We applied tDCS to M1 only. This may be why differential effects were not seen across groups for the long transfer test.

Participants were asked to rate whether they felt sensations during the tDCS, and to rate the severity of sensations. We found that more participants in the C-M1 group reported tingling and discomfort on Day 1, relative to the A-M1 and sham groups. This effect was only seen on Day 1. While this may be a significant finding, performance did not differ across the three groups on Day 1. Therefore, the greater reports of tingling and discomfort did not seem to negatively influence performance or learning.

Participants were asked to guess which tDCS group they thought they were in. We found that more participants in the C-M1 group correctly guessed their stimulation condition on the first day only. Performance was not different across the three groups on day 1, thus, correctly guessing the stimulation group may not have had an effect on performance.

## 5. Limitations

One limitation of this study is that for feasibility, this study was a between-subjects, rather than a within-subjects, design. A within-subject, cross-over design could be employed, to minimize the effects of individual differences on the learning process. However, pilot data suggested that it was difficult to create variations of the golf putting task, even with a washout period. A second limitation is that we used a relatively small sample of convenience, recruited from the University of Toronto campus and did not conduct a power analysis; together this limits our ability to generalize the findings. A third limitation is that we used a fixed-dose of tDCS. Effects of tDCS are often variable across individuals due to factors such as skull and brain anatomy [42,43]. Future studies should aim to utilize current flow modelling to personalize the dose of tDCS. This would require magnetic resonance scans from each participant, but it would also enable researchers to standardize the amount and direction of current reaching relevant brain areas [22,44]. A fourth limitation is that we were not powered to perform sex and gender-based analyses. However, all data are made available in the raw data file in the supplementary material. Finally, a fifth limitation is that we did not record neurophysiological data (e.g., motor evoked potentials (MEPs), or electroencephalogram (EEG)). Future studies should look to record neurophysiological measures, such as MEPs, to observe whether tDCS induces the expected changes in corticospinal excitability and the impact of training on excitability.

## 6. Conclusions

Priming tDCS did not affect motor skill acquisition, however, C-M1 tDCS priming led to better retention of performance, suggesting a potential facilitative effect on motor learning, defined as retention of performance, of a naturalistic motor task. Our findings partially support the BCM rule of metaplasticity and suggest it may be possible to improve learning using C-M1 tDCS priming before task practice in a sports context.

## Supporting information

S1 File Current flow modelling image. (DOCX)

S2 File Raw data file. (XLSX)
