# Supplementary material for: Priming the primary motor cortex with transcranial direct current stimulation: Effect on learning the golf putt
Source: PLoS One. 2025 May 28;20(5):e0324983. doi: 10.1371/journal.pone.0324983 (PMC12118825; doi:10.1371/journal.pone.0324983)
Supplement: S1 File — (DOCX) [file pone.0324983.s001.docx]

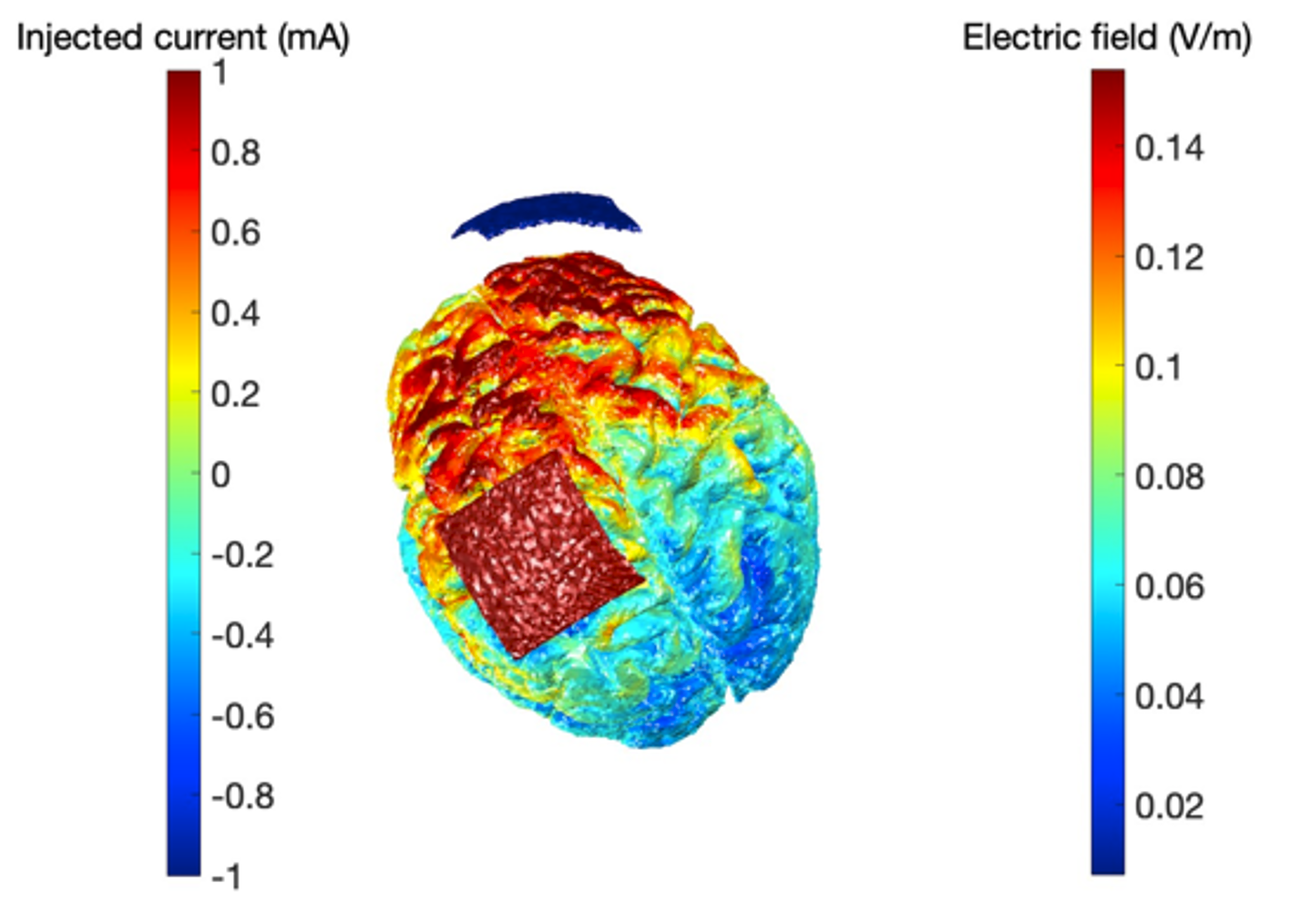


**Supplementary Figure 1. Current Flow Modeling.** To confirm that the electrode placement would result in current flowing through the desired region of left M1, current flow modelling was used. The software package ROAST was used. A sample MRI structural T1 scan was used to create a model of the e-fields generated in the brain through tDCS.
